# Supplementary material for: Dynamics in the Phytophthora capsici Effector AVR3a11 Confirm the Core WY Domain Fold
Source: Biochemistry. 2025 Feb 20;64(5):1146–56. doi: 10.1021/acs.biochem.4c00660 (PMC11883810; doi:10.1021/acs.biochem.4c00660)
Supplement: Supplementary file 1 — bi4c00660_si_001.pdf [file bi4c00660_si_001.pdf]

Supporting Information

Dynamics in the *Phytophthora capsici* effector AVR3a11 confirm the core WY domain fold

James Tolchard[1#], Vicki S. Chambers[1$], Laurence S. Boutemy[2£], Mark J. Banfield[2] and Tharin M. A. Blumenschein[1]*

[1]School of Chemistry, Pharmacy and Pharmacology, University of East Anglia, Norwich Research Park, Norwich, NR4 7TJ, UK.

[2]Department of Biochemistry and Metabolism, John Innes Centre, Norwich Research Park, Norwich, NR4 7UH, UK.

Present Addresses

[#]Centre de RMN Très Hauts Champs de Lyon (UMR5082—CNRS, ENS Lyon, UCB Lyon 1), Université de Lyon, 69100 Villeurbanne, France.

[$]Illumina, Illumina Centre, Cambridge, CB216DF, UK.

[£]Norwich School, Norwich, NR1 4DD, UK.

* Email: t.blumenschein@uea.ac.uk

KEYWORDS: effector protein, hydrogen-deuterium exchange, NMR, dynamics, oomycete

Figures

Figure S1. Two-dimensional [^{1}H, ^{15}N]-HSQC NMR spectra of AVR3a11$_{63\text{-}132}$ at different temperatures: 300 K (black, full contours), 295 K (navy, single contour), 290 K (purple, single contour), 285 K (red, single contour), 280 K (blue, single contour), and 275 K (green, full contours). New peaks that appeared with the decrease in temperature are labelled with red boxes, and the peak that disappeared is labelled with a blue box.

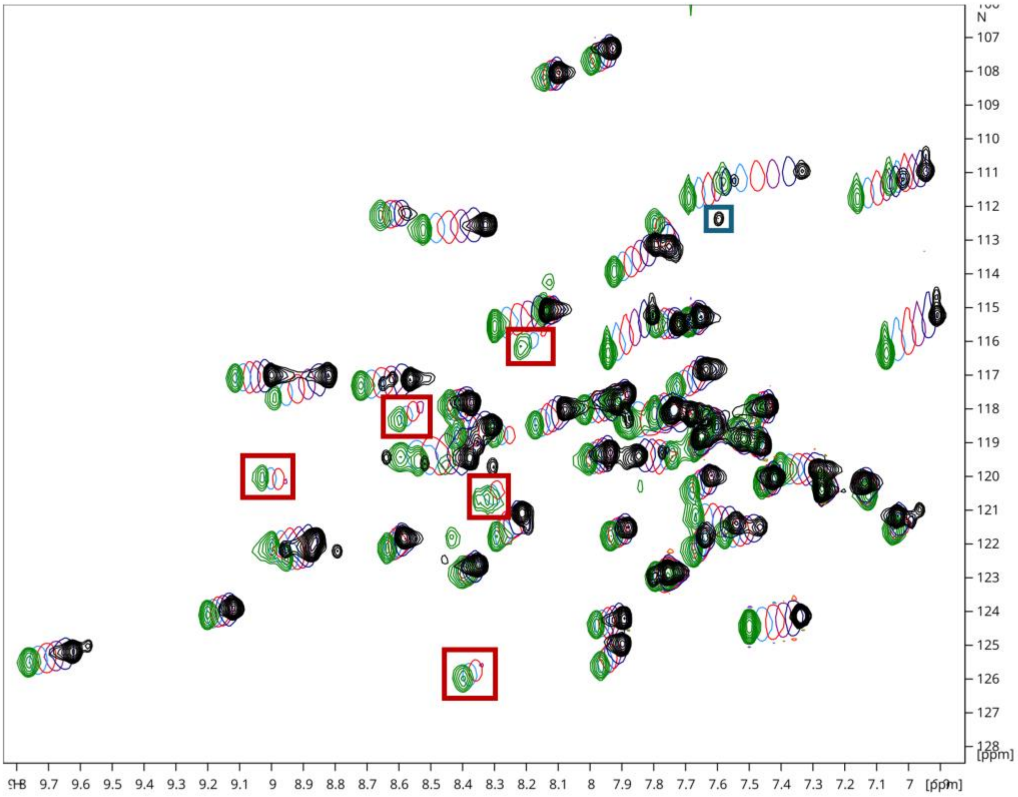

Figure S2. AVR3a11$_{63\text{-}132}$ sequence coloured by assignment status: fully assigned (dark green), side chain assigned but not backbone atoms (light green), partially assigned (orange) and not visible in the spectra (grey).

GLTDLFKTEK AAVKKMAKAI

MADPSKADDV YQKWADKGYT

LTQLSDFLKS KTRGKYDRVY

NGYMTYRDYV

Figure S3. Detail of regions in the [^{1}H, ^{15}N]-HSQC two-dimensional NMR spectrum of AVR3a11$_{63\text{-}132}$, showing small peaks corresponding to minor conformations of AVR3a11$_{63\text{-}132}$ for Lys111 (top panel), Lys 80 (middle panel), Lys72 and Lys 76 (bottom panel). All peaks were assigned with the aid of three-dimensional triple resonance spectra.

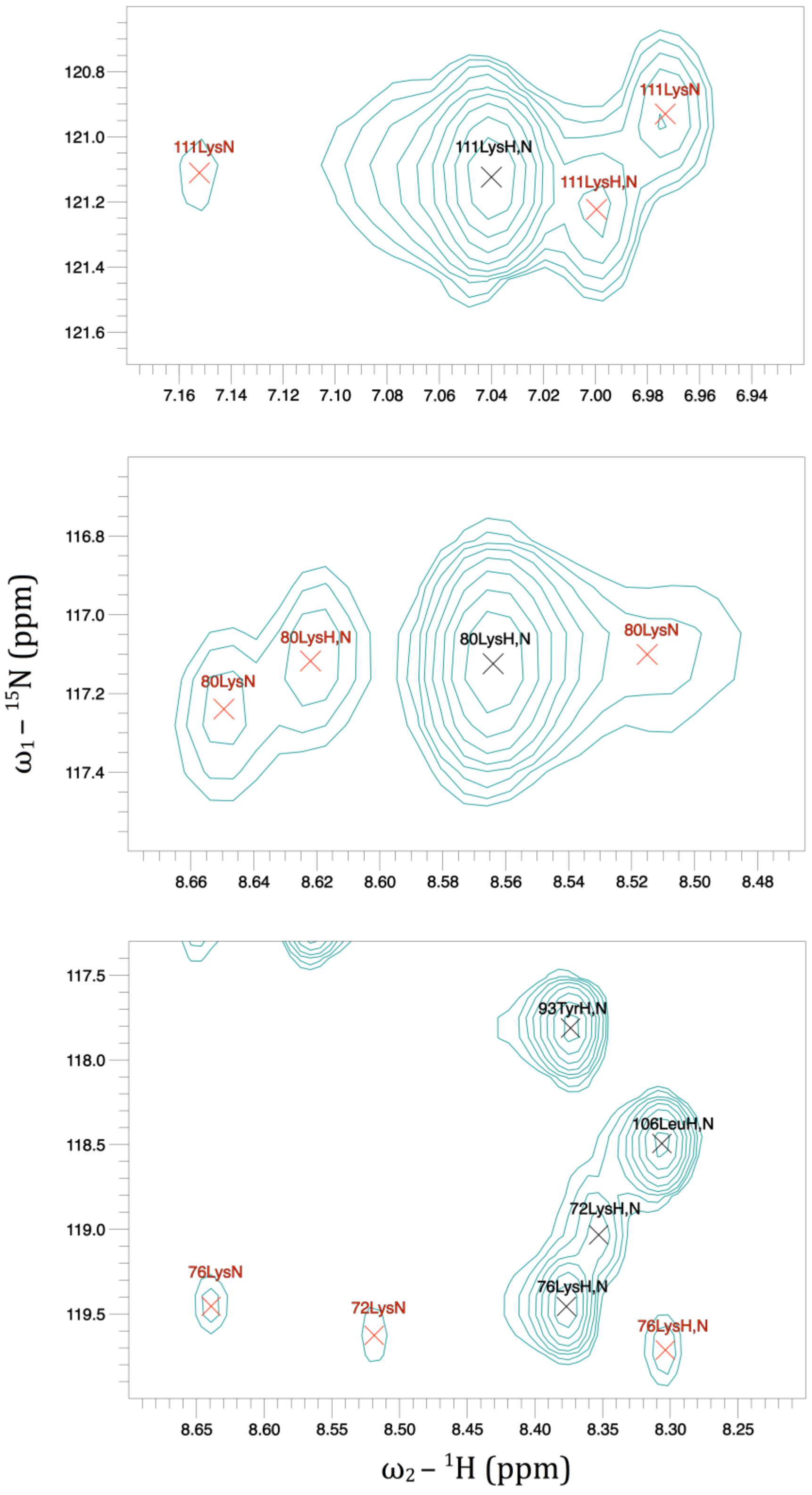

Figure S4. Model-free analysis of AVR3a11$_{70\text{-}132}$ relaxation plotted against residue number, displaying the order parameter S^2 (top), effective correlation time for internal motions τ_e (middle), and exchange rate R_{ex} (bottom). Data points are coloured by the model used to fit each residue, in which M2 (white) uses S^2 and τ_e, M3 (black) uses S^2 and R_{ex}, M4 (grey) uses S^2, τ_e and R_{ex}, and M5 (striped) uses S_s^2, S_f^2, τ_e and R_{ex}, where S_s^2 and S_f^2 are, respectively, slow and fast order parameters. Two residues (Ala79 and Leu106) fitted as M5 yielded very large, inaccurate τ_e values that were excluded from the plot. Error bars represent the fit error, and are too small to be seen in the order parameter plot.

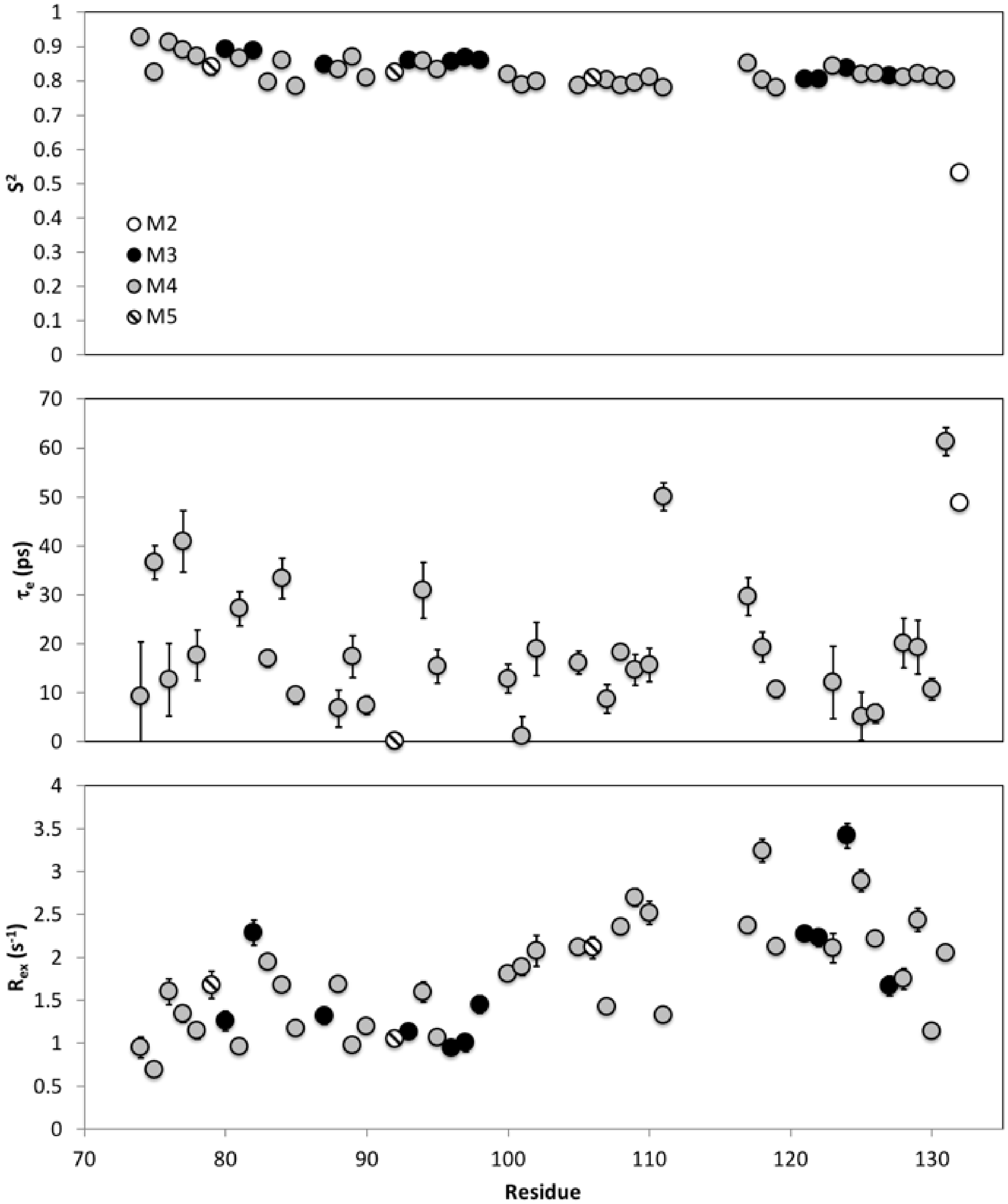

Figure S5. Reduced spectral density analysis for AVR3a11$_{70\text{-}132}$ plotted against residue number. Frequencies analysed correspond to slow [J(0), top panel], intermediate [J(ω_N), middle panel] and fast [J(ω_H), bottom panel] motions, calculated from data at 500 MHz (black circles) and 800 MHz (white squares).

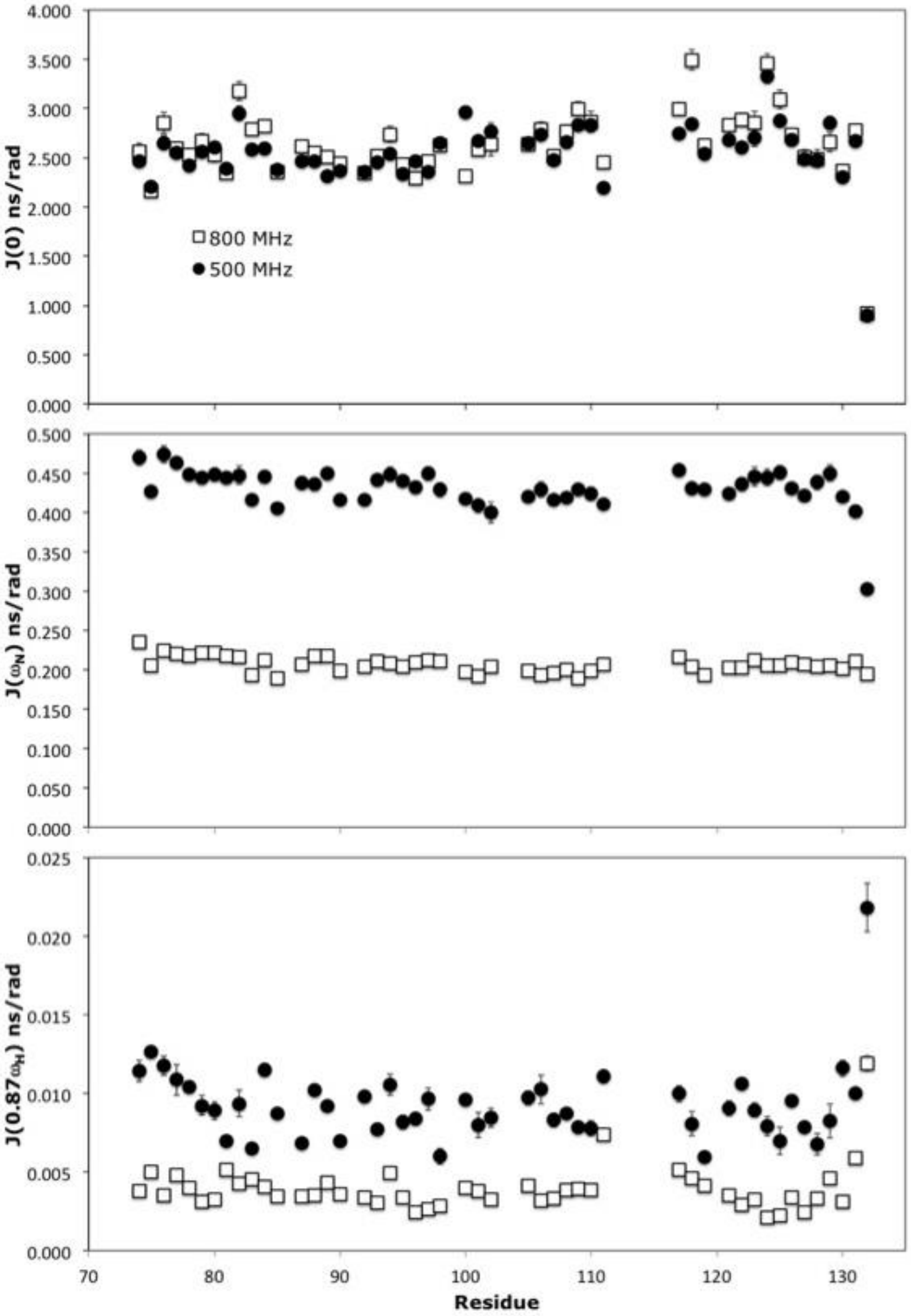

Figure S6. Correlation between J(0) calculated from data at 500 MHz and 800 MHz, to confirm consistency between the two data sets. Residues 100 and 118 show the largest differences in J(0) values.

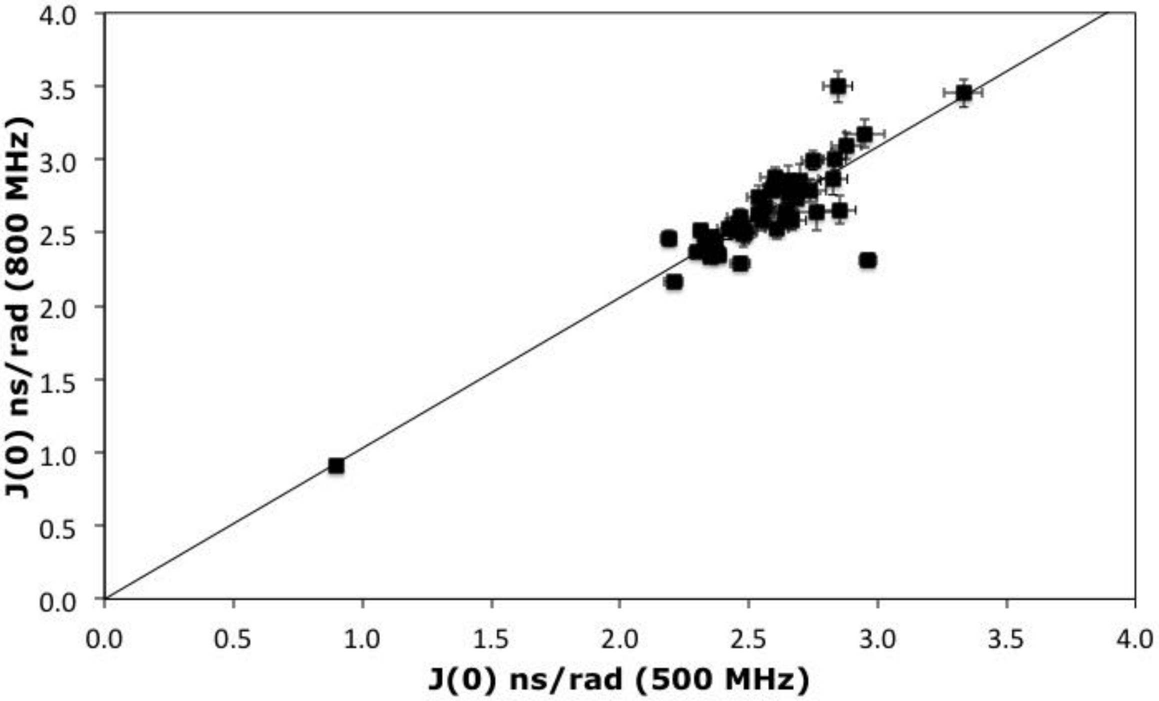

Figure S7. Most representative conformer of AVR3a11$_{63\text{-}132}$ solution structure, coloured according to evidence for conformational exchange: residues with peaks broadened beyond detection in the [^{1}H, ^{15}N]-HSQC NMR spectrum (grey) and residues with minor peaks in the [^{1}H, ^{15}N]-HSQC NMR spectrum, corresponding to alternative conformations (blue). Residues which do not show evidence of conformational exchange are coloured in green.

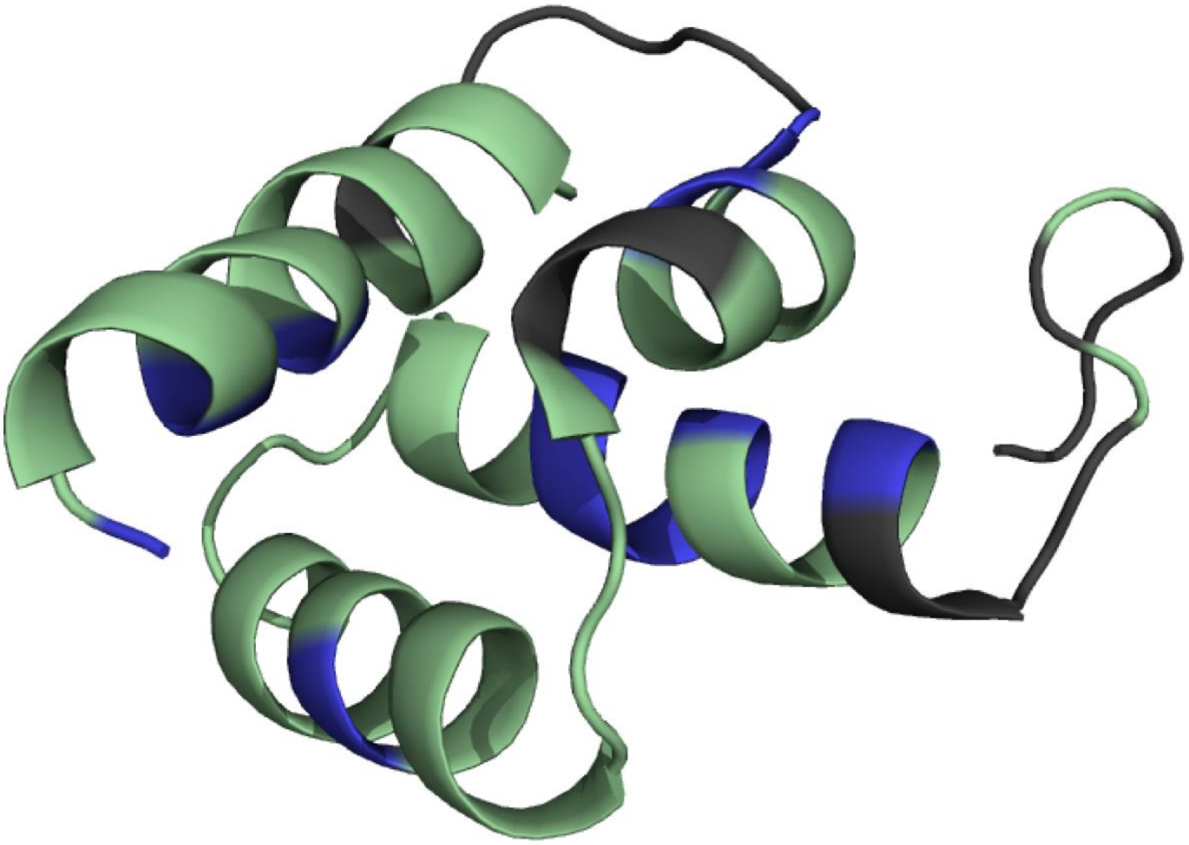

Table S1. Experimental restraints and structural statistics for AVR3a11$_{63-132}$.

**Distance restraints**	
All	870
Intra-residue	158
Sequential	227
Medium range ($1 < \|i - j\| \leq 4$)	296
Long range ($\|i - j\| > 4$)	195
**Dihedral angle restraints**	98
**Residual constraint violations**	
NOE violations > 0.2 Å	0
Dihedral angle violations > 5°	0
Van der Waals violations > 0.1 Å	0
**Backbone deviation from average structure (RMSD)[a]**	
All residues	2.9 ± 0.7 Å
Ordered	0.75 ± 0.12 Å
**Ramachandran plot[a,b]**	
Most favoured regions	94.40%
Additionally allowed regions	5.60%
Generously allowed or disallowed regions	0.00%

[a]Calculated using the validation software PSVS [47].
[b]Ordered residues, calculated using Procheck [49].
